# Timing of transcranial direct current stimulation (tDCS) combined with speech and language therapy (SLT) for aphasia: study protocol for a randomized controlled trial

**DOI:** 10.1186/s13063-022-06627-9

**Published:** 2022-08-17

**Authors:** Sameer A. Ashaie, Samantha Engel, Leora R. Cherney

**Affiliations:** 1 Center for Aphasia Research and Treatment, Shirley Ryan AbilityLab, 355 E. Erie St, Chicago, IL 60611 USA; 2 Physical Medicine and Rehabilitation, Feinberg School of Medicine, Northwestern University, Chicago, IL USA; 3 Communication Sciences and Disorders, Northwestern University, Evanston, IL USA

**Keywords:** Aphasia, Transcranial direct current stimulation, tDCS, Speech-language therapy, Stroke, Anodal, Cathodal, Randomized controlled trial

## Abstract

**Background:**

Studies suggest that language recovery in aphasia may be improved by pairing speech-language therapy with transcranial direct current stimulation. However, results from many studies have been inconclusive regarding the impact transcranial direct current stimulation may have on language recovery in individuals with aphasia. An important factor that may impact the efficacy of transcranial direct current stimulation is its timing relative to speech-language therapy. Namely, online transcranial direct current stimulation (paired with speech-language therapy) and offline transcranial direct current stimulation (prior to or following speech-language therapy) may have differential effects on language recovery in post-stroke aphasia. Transcranial direct current stimulation provided immediately before speech-language therapy may prime the language system whereas stimulation provided immediately after speech-language therapy may aid in memory consolidation. The main aim of this study is to investigate the differential effects of offline and online transcranial direct stimulation on language recovery (i.e., conversation) in post-stroke aphasia.

**Methods/design:**

The study is a randomized, parallel-assignment, double-blind treatment study. Participants will be randomized to one of four treatment conditions and will participate in 15 treatment sessions. All groups receive speech-language therapy in the form of computer-based script practice. Three groups will receive transcranial direct current stimulation: prior to speech-language therapy, concurrent with speech-language therapy, or following speech-language therapy. One group will receive sham stimulation (speech-language therapy only). We aim to include 12 participants per group (48 total). We will use fMRI-guided neuronavigation to determine placement of transcranial direct stimulation electrodes on participants’ left angular gyrus. Participants will be assessed blindly at baseline, immediately post-treatment, and at 4 weeks and 8 weeks following treatment. The primary outcome measure is change in the rate and accuracy of the trained conversation script from baseline to post-treatment.

**Discussion:**

Results from this study will aid in determining the optimum timing to combine transcranial direct current stimulation with speech-language therapy to facilitate better language outcomes for individuals with aphasia. In addition, effect sizes derived from this study may also inform larger clinical trials investigating the impact of transcranial direct current stimulation on functional communication in individuals with aphasia.

**Trial registration:**

ClinicalTrials.gov NCT03773406. December 12, 2018.

## Background

Aphasia is an acquired (typically left-hemisphere stroke) multi-modality disturbance of language that impacts around 2 million people in the USA and is more common than disorders such as Parkinson’s disease and muscular dystrophy [1]. The disorder impacts not only comprehension and production of oral language but also reading and writing. The consequences of aphasia extend beyond the language impairment and can negatively impact all aspects of the person’s daily life including social, vocational, and recreational activities. Currently, speech-language therapy (SLT) is the most effective way to treat aphasia [2]. However, even if SLT is intensive, individuals with aphasia are left with residual language impairments that impact participation and quality of life. Recent research suggests that language recovery in individuals with aphasia can be boosted by pairing SLT with a non-invasive, safe, and low-cost form of brain stimulation known as transcranial direct current stimulation (tDCS) [3].

In conventional tDCS, direct current is applied to the scalp via two electrodes: an anode and a cathode. tDCS does not induce neuronal firing but modulates the resting membrane potential of the neurons which can then impact the rates at which neurons fire [4]. Early research in animals and human motor cortex established that tDCS effects are polarity specific [4, 5]. Anodal stimulation may increase cortical excitability due to neuronal depolarization while cathodal stimulation decreases cortical excitability due to neuronal hyperpolarization [5].

### tDCS polarity in aphasia

In tDCS studies of aphasia, anodal tDCS is understood to improve performance by increasing cortical excitability while cathodal tDCS impairs performance by decreasing cortical excitability [6, 7]. Therefore, many studies [8–10] have applied anodal tDCS to the left-hemisphere perilesional areas under the assumption that it will upregulate the perilesional areas and improve performance. Other studies [11, 12] have applied cathodal tDCS to homologous right-hemisphere areas under the hypothesis that these areas are maladaptive for language and that downregulating them allows the perilesional left-hemisphere area to process language more efficiently [13].

The notion that anodal tDCS improves performance whereas cathodal tDCS impairs performance is derived primarily from tDCS studies of the motor cortex, and such inferences may not be applicable to language and cognition [14]. The assumption that cathodal tDCS impairs performance is challenged by studies [15–17] that find performance improvements following cathodal tDCS to the left hemisphere. For example, Cherney and colleagues [18] found that cathodal tDCS led to improvement in behavioral measures similar to that of anodal tDCS and significantly more than sham in individuals with aphasia. Moreover, they also found that tDCS to the left hemisphere resulted in an increase in activation of voxels in both hemispheres with more activation in the left perilesional areas. However, studies have differed in terms of stimulation parameters (e.g., timing of tDCS and stimulation intensity). These variations may differentially impact how anodal and cathodal tDCS affect language recovery.

### Stimulation site, intensity, and duration

There is no consensus on the optimal site for stimulation in tDCS studies of aphasia [6]. Some studies [15, 19, 20] have targeted ipsilesional inferior frontal gyrus (IFG) while other studies [11, 21] have targeted contralesional right IFG. Furthermore, other studies have targeted ipsilesional left posterior superior temporal gyrus (pSTG) [9, 12] or contralesional right pSTG [12]. More recently, the cerebellum has also been a site of interest [22, 23]. Most studies have stimulation intensities of 1mA or 2mA with current densities between .029 and 08mA/cm^2^ [6, 24]. The stimulation duration of tDCS studies for aphasia has varied with the majority of studies stimulating for 20 min [6].

### Timing of stimulation

Another critical factor that could determine the efficacy of tDCS is the timing of stimulation and the brain state [25–27]. Studies in healthy individuals have shown that applying offline cathodal tDCS improves behavioral and motor performances [27, 28]. These findings have been explained by the Bienenstock-Cooper-Munro (BCM) principle of homeostatic plasticity [27–29]. According to the BCM theory of “sliding-threshold” for bi-directional plasticity, initial low synaptic activity lowers the threshold for long-term potentiation (LTP) like plasticity [29, 30]. Thus, cathodal tDCS may lower the threshold for LTP-like plasticity during SLT which may then enhance language recovery [26]. On the other hand, online cathodal tDCS could improve behavioral performance by suppressing neuronal noise and enhancing the relevant neuronal signal [26]. Thus, if we want to maximize the impact of tDCS as an adjuvant to SLT, the role of timing and brain state needs to be considered.

It is presumed that the impact of tDCS is maximized when paired simultaneously with SLT (online tDCS). However, tDCS provided immediately before SLT (offline-before SLT) may also impact language recovery since it primes the language system at rest for subsequent language rehabilitation. It is also possible that tDCS after SLT (offline-after SLT) will aid in language recovery because it helps in memory consolidation and reconsolidation. The question of whether online tDCS and offline tDCS impact language recovery after post-stroke aphasia differentially has not been investigated to date.

## Aims

The main aim of the study is to investigate the differential effects of offline and online tDCS on language recovery in post-stroke aphasia. Specifically, we plan to conduct a randomized clinical trial (*N* = 48) in individuals with aphasia that compares the differential effects of tDCS delivered prior to, concurrent with, and following SLT.

## Method/design

### Study design

**Fig. 1:**
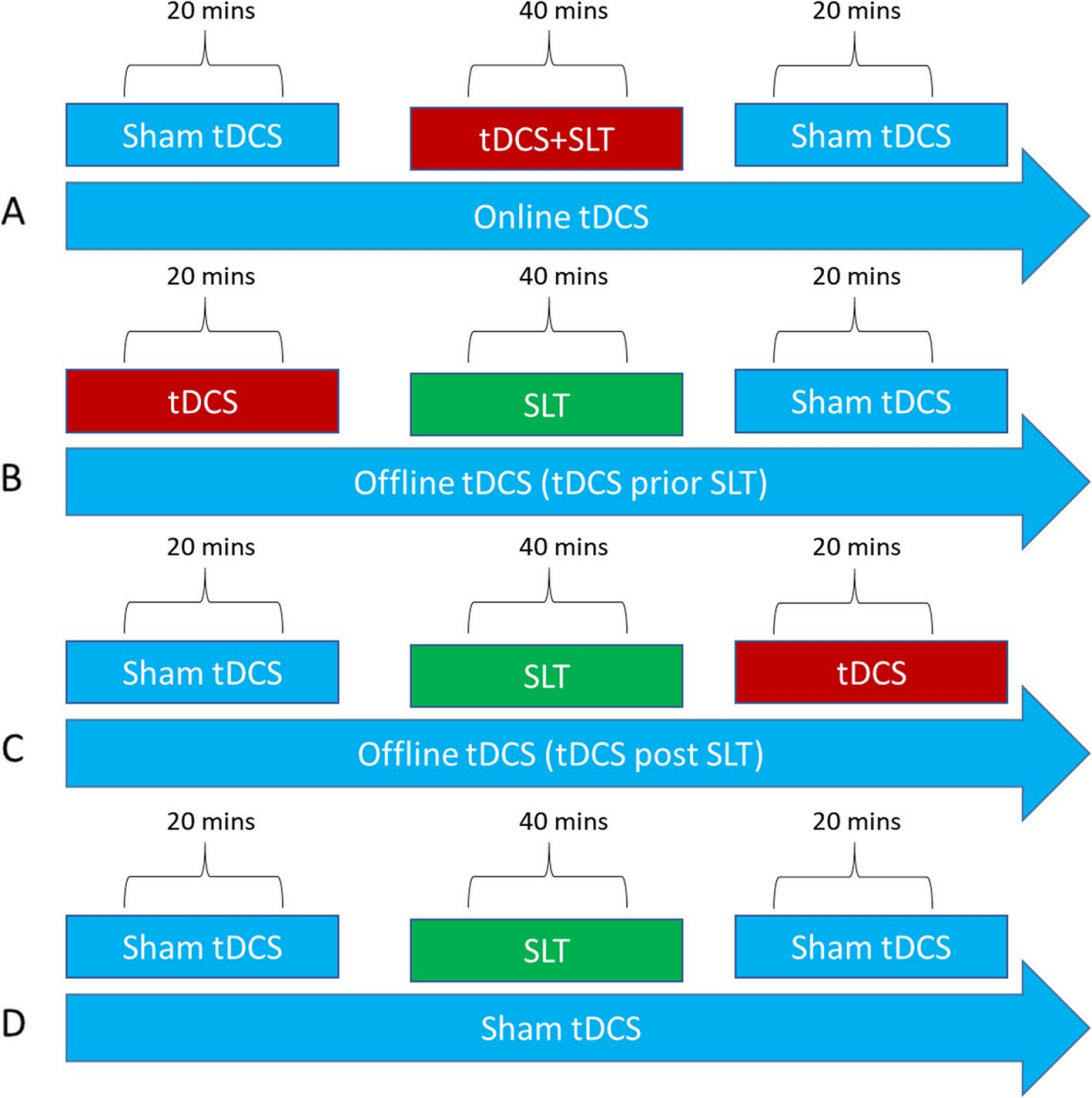
Treatment timeline by tDCS condition. **A** tDCS concurrent with the first 20 min of SLT, **B** tDCS prior to SLT, **C** tDCS post-SLT, and **D** sham tDCS (only SLT)

This is a randomized, parallel-assignment, double-blind exploratory treatment study. The exploratory randomized clinical trial will be conducted with 48 individuals with aphasia. Participants will be randomized to one of four treatment conditions (see below), with 12 participants per group. Participants will receive treatment 5 days a week for 3 weeks (i.e., 15 sessions). All participants receive 40 min of SLT. To equalize session length and maintain blinding, participants will also receive an additional 20 min of sham tDCS (see Fig. 1). The four treatment groups are as follows:

1. Offline tDCS-before therapy (tDCS prior to SLT) — participants will receive 20 min of tDCS followed by 40 min of SLT. The session will end with 20 min of sham tDCS.
2. Online tDCS (tDCS concurrent with SLT) — participants will receive sham tDCS for 20 min followed by concurrent SLT and tDCS. tDCS will be applied for the first 20 min of the 40-min SLT session. The session will end with 20 min of sham tDCS.
3. Offline tDCS-after therapy (tDCS after SLT) — participants will receive sham tDCS for 20 min followed by 40 min of SLT. The session will end with 20 min of tDCS.
4. Sham tDCS (only SLT) — participants will receive 20 min of sham tDCS followed by 40 min of SLT. The session will end with 20 min of sham tDCS. The sham tDCS group will serve as a control group and allow us to investigate the impact of SLT without tDCS.

Subjects will be requested to come for a baseline session (3 to 4 h) before the treatment intervention begins. The baseline assessments may be done over two visits. In the baseline session, aphasia type and severity will be determined by the Western Aphasia Battery Revised (WAB-R) [31]. The WAB-R will be administered by a trained speech-language pathologist who is blind to the treatment group assignment. Participants will also perform baseline tasks of language and cognition in this session to further characterize their language and cognitive status.

**Fig. 2:**
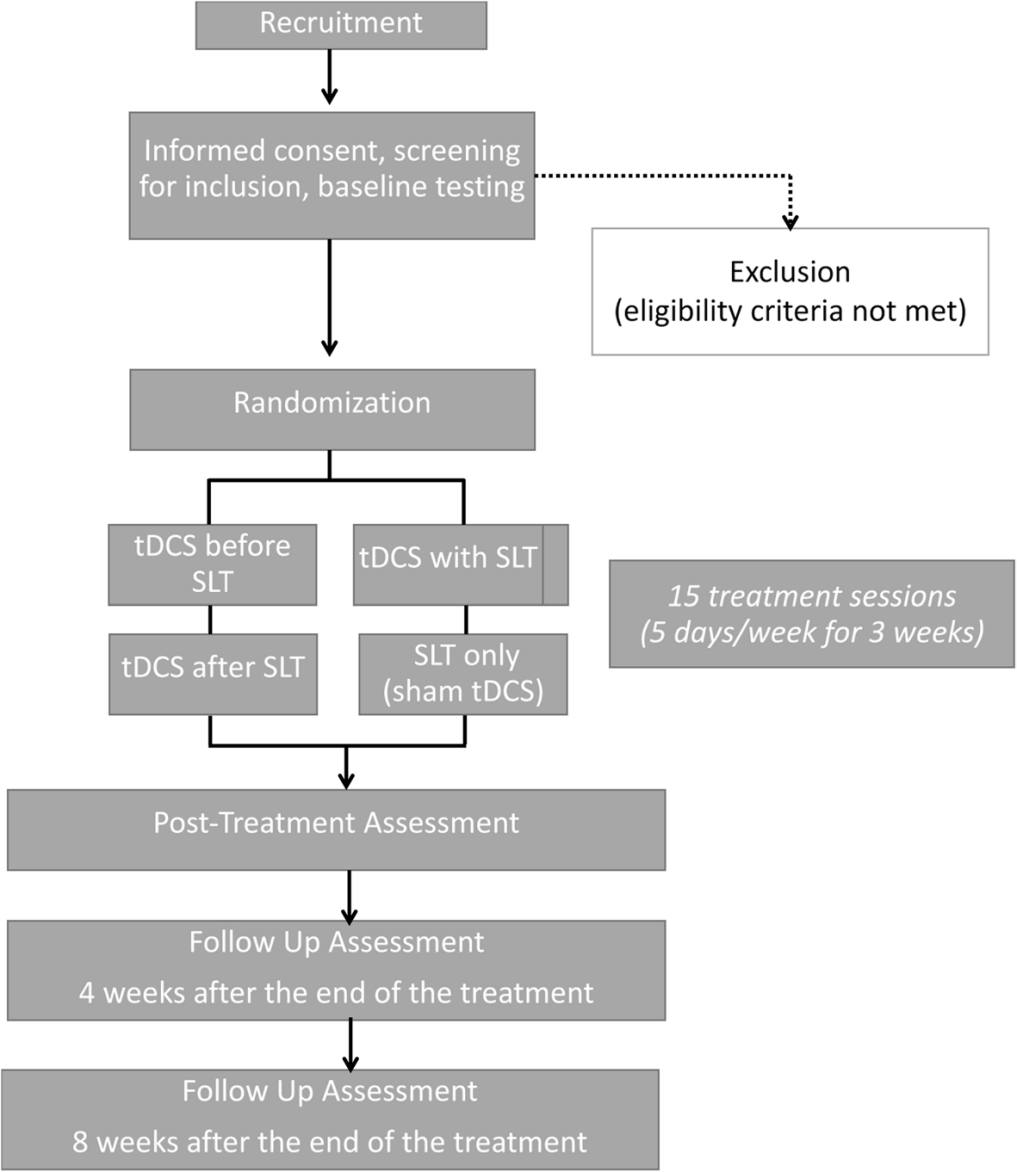
Flowchart of the study timeline. Schematic diagram of the study protocol

Following the baseline assessment, all subjects who satisfy the inclusion criteria will participate in 3 weeks of the treatment intervention (i.e., 15 sessions). Subjects will be asked to return for an assessment immediately following the end of treatment and for follow-up testing 4 and 8 weeks after the end of treatment. The study schedule and assessments are shown in Table 1 and Fig. 2.

**Table 1:** Trial schedule of enrollment, intervention, and assessments

	Study period					
	Enrollment	Allocation	Treatment intervention	Post-treatment assessment	Follow-up assessment	
Timepoint	***−t***_***1***_	0	***t***_***1***_	***t***_***2***_	***t***_***3***_	***t***_***4***_
**Enrollment**						
**Eligibility screen**	X					
**Informed consent**	X					
**Allocation**		X				
**Interventions**						
***Offline tDCS-before SLT***			X			
***Online tDCS-during SLT***			X			
***Offline tDCS-after SLT***			X			
***Sham tDCS-SLT only***			X			
**Assessments**						
***Western Aphasia Battery-Revised***	X			X	X	
***Wechsler Memory Scale-III***	X			X	X	
***Picture Description from Comprehensive Aphasia Test***	X			X	X	
***Apraxia of Speech Rating Scale***	X			X	X	
***Modified Rankin Scale***	X			X	X	
***Center for Epidemiologic Studies Depression Scale***	X			X	X	
***Communication Confidence Rating Scale For Aphasia (CCRSA)***	X			X	X	
***The Stroke and Aphasia Quality of Life Scale-39-Item Version (SAQOL-39)***	X			X	X	
***Philadelphia Naming Test***	X			X	X	
***Arizona Semantic Test***	X			X	X	
***Conners’ Continuous Performance Test II (CPT-II)***	X			X	X	
***Demographic data***	X					
***List of medications***	X

### Setting

The study will be conducted at the Shirley Ryan AbilityLab, a rehabilitation hospital located in Chicago, IL.

### Ethical approval and trial registration

The study has been approved by Northwestern University IRB (STU00204900). The study is registered on the ClinicalTrials.gov website (ID: NCT03773406).

### Participants and recruitment

Flyers will be posted throughout the Shirley Ryan AbilityLab so that prospective subjects can contact the Center for Aphasia Research and Treatment if they are interested. Flyers will be sent to local speech-language pathologists and physicians so that potential participants can be informed. Participants from our previous studies who have given us permission to contact them regarding future studies will also be contacted. Interested participants can contact the Center for Aphasia Research and Treatment. Study personal will keep in regular contact with participants and their caregivers through phone/e-mail to ensure study retention. The study will be conducted in person at the research lab, thus ensuring adherence to intervention protocols. All protocol changes will be approved by the IRB. Participants cannot participate in other language treatment or brain stimulation intervention studies while participating in this study.

### Inclusion criteria

Participants will be included if they meet the following criteria: (a) diagnosis of fluent or non-fluent aphasia subsequent to a left-hemisphere infarct(s) confirmed by MRI or CT scan; (b) Aphasia Quotient on the WAB-R of 35–85; (c) at least 4 months post-onset of aphasia (this is beyond the stage of spontaneous recovery); (d) 18–80 years of age; (e) premorbidly fluent in English; (f) premorbidly right-hand dominant per the Edinburg Handedness Inventory; (g) visual acuity of 20/40 corrected; (h) auditory acuity no worse than 30 dB HL on pure tone testing, aided in the better ear; and (i) education greater than 12th grade.

### Exclusion criteria

Participants will be excluded if they have any other neurological condition (other than cerebral vascular disease) that could impact language and cognition such as Alzheimer’s disease, Parkinson’s disease, primary progressive aphasia, and traumatic brain injury. Participants will also be excluded if they have active substance use disorder or epilepsy.

### Sample size and power

The study will enroll a total of 48 subjects (12 in each group). Power calculations were based on our previous work with similar participants, and planned two-tailed analyses (*α* = .05) for the primary outcome measure, the simulated conversation on the trained script.

In preliminary work without tDCS, we observed improvement in trained script accuracy at the end of language treatment relative to baseline was 10.4% (SD = 8.2%). Assuming similar variability in the proposed study, *n* = 12 subjects per group will allow us to estimate changes in accuracy with precision (half-width of a 95% confidence interval) of ±5.2% in each group. In addition, a sample size of *n* = 12 subjects per group will provide 80% power with two-sided *α* = 0.5 for a two-sample *t*-test to detect a 9.9% difference in accuracy improvement between treatment arms and the sham treatment (e.g., improvement in accuracy of 10.4% and 20.3% in the two arms, respectively).

### Randomization

Participants will be randomized to each of the four treatment arms, i.e., four combination treatment sequences (tDCS prior to SLT, tDCS concurrent with SLT, tDCS following SLT, or sham tDCS (SLT only)). Randomization will be stratified by the baseline WAB-R AQ score (<60 vs. ≥60), and randomization will be conducted using the permuted block method. Randomization will be set by a statistician.

### Consent process

Informed consent will be obtained in a private office by a trained clinician/researcher who has experience with communicating with individuals with aphasia. They will ensure that subjects understand the purpose of the study, the procedures, and minimal risk and potential benefits associated with the study. The subjects will be made aware of their rights as research subjects (e.g., withdrawing at any point during the study without consequences). Subjects can take the consent forms home to review and consult with their family members prior to signing the consent forms.

## Intervention

### tDCS intervention

**Fig. 3:**
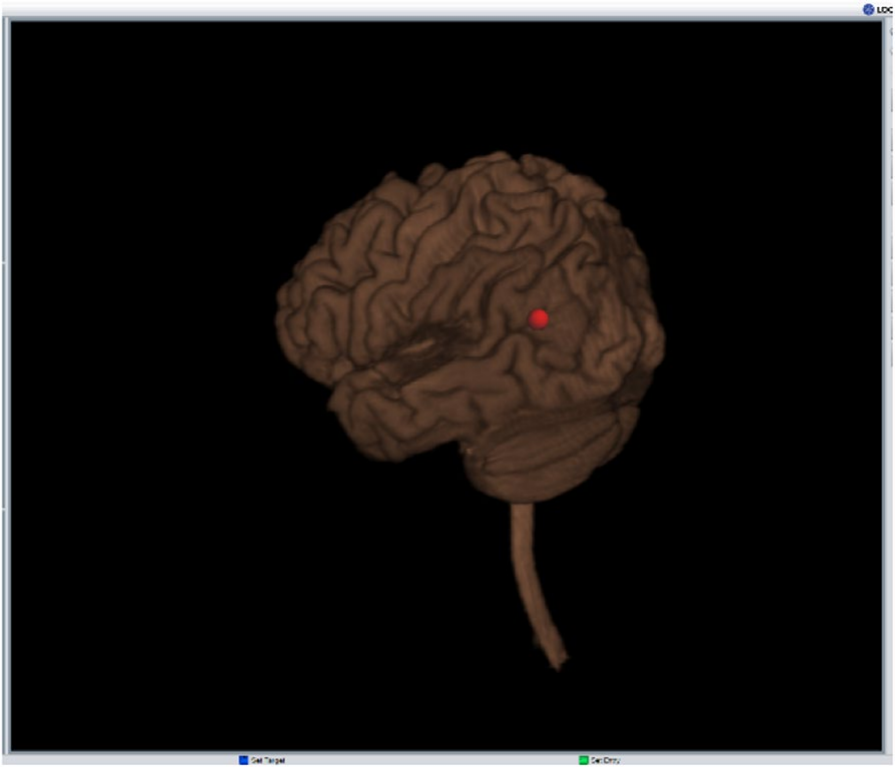
Example of target electrode location. Subjects’ heads are co-registered with MR images in a 3-D space to identify target location for the tDCS stimulation site. The red dot denotes the location of the target tDCS electrode

*tDCS parameters and intensity*: We will use 2mA of current intensity because it will yield higher current densities than 1mA or 1.5mA and may result in larger behavioral effects [6, 32]. *Duration*: Based on the duration of tDCS applied in previous studies [19, 24, 33], we will administer tDCS for 20 min. *Polarity*: There is no clear evidence that anodal tDCS improves behavioral performance while cathodal tDCS impairs behavioral performance [6, 14, 15]. However, a recent tDCS study [16] has shown that left-hemisphere cathodal tDCS elicits the most consistent language improvement in persons with aphasia. Thus, we will use left-hemisphere cathodal tDCS in our study. *Stimulation site*: There is no consensus on the optimal cortical site for tDCS in PWA [6, 24]. The choice for optimal site is complicated by lesion location and size and inter-individual differences in functional language reorganization post-stroke [6]. Recent advances have resulted in creating individualized montages based on factors such as current flow [34] and language outcomes prior to the treatment [16]. However, such methods are costly and time consuming and not viable if tDCS is seen as a low-cost adjuvant to language therapy. To circumvent this problem, we will target the left angular gyrus (AG) for stimulation. The AG has been implicated in multiple functions such as semantic processing, reading and comprehension, attention, and memory retrieval [35]. It is located at the junction of occipital, temporal, and parietal lobes [35]. Connectivity analyses have shown that AG is a hetero-modal hub linking language and episodic memory areas in the frontal, parietal, and temporal lobes as well as sensory and motor areas [36–38]. The rich functional and anatomical connectivity of AG with other brain regions and its role as a hetero-modal hub make it an ideal site for tDCS stimulation [39]. Stimulating a hub can increase functional connectivity with other brain regions and maximize the impact of tDCS [39]. It is possible that some persons with aphasia may have complete damage to the left AG and cannot be targeted by tDCS. To circumvent this problem, we will target the tissue area most proximal to the left AG. *tDCS implementation*: We will deliver 2mA of direct current for 20 min using a constant current stimulator (Soterix Medical., New York, USA) via a 15-cm^2^ saline-soaked sponge. To stimulate the left AG, a 15-cm^2^ cathodal electrode will be placed over CP5 according to the 10–20 international EEG system for electrodes. The electrode will be secured in position by a custom-built EEG cap http://www.easycap.de/ that will be marked with the location for AG. We will then confirm that the cathodal electrode is over left AG and not dead tissue through the use of a neuronavigation system (eXimia 3.1, Nexstim Ltd., Helsinki, Finland). The neuronavigation system allows us to co-register subjects’ heads in 3-D space with their T1-weighted MR images. The neuronavigation system identifies the scalp coordinates on the cortex and then maps the computerized picture of the scalp to the physical location on the person (see Fig. 3). Since tDCS requires two electrodes, the anodal “return” electrode of 25cm^2^ will be placed on the center of the supraorbital region. A larger return electrode was chosen to lessen its effect since recent studies have indicated that the return electrode may also provide some electrical stimulation to the cortex [35] .

At the beginning of the tDCS, the current will be slowly increased (ramp-up) during the first 30 s until it reaches 2mA. The ramp-up process will allow the participants to acclimate to tDCS-induced sensations (e.g., itching). At the end of the tDCS session, the current will be slowly ramped-down in the last 30 s to 0mA. The total duration of the 2-mA direct current will be maintained for 20 min.

For the sham tDCS, the tDCS stimulation will be given for 30 s and then the tDCS device will be turned off. The 30-s stimulation will produce tDCS-induced sensations so that participants will not be aware when the tDCS is turned off.

### Language treatment

**Fig. 4:**
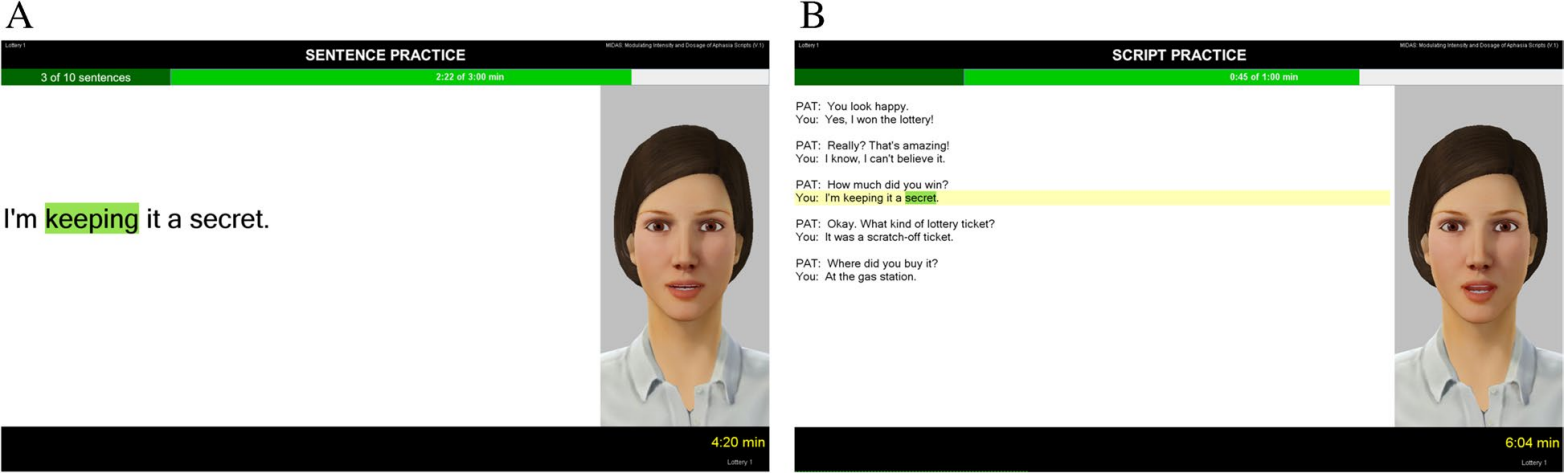
The AphasiaScripts software. **A** Example of a single conversational sentence. The highlighted word reflects the word that is being read aloud by the participant. **B** Example of a full conversation that participant practices with PAT (virtual therapist) and YOU (participant). Highlighted sentences reflect what the participant is practicing

We will use *a standard-of-care treatment — script training —* to provide language therapy to the subjects [40–42]. The scripting treatment will be delivered by a computer program that provides script training in an interactive conversational context. Script training involves participants repeatedly practicing (e.g., pointing and choral reading) sentences within different contexts as either a dialogue or a monologue. The advantage of using *AphasiaScripts®*, a computer program with a virtual therapist instead of a human therapist, is that it removes extraneous variables associated with the human therapist. For example, it is possible that a human therapist might show differential levels of encouragement to different subjects and thus impact treatment fidelity. However, a virtual therapist will be consistent in providing the speech-language therapy across participants. Additionally, the program tracks every response and records choral and independent verbal productions that can be scored later by an unbiased clinician. Scripts will be ten sentences long and developed from common templates on the topics of buying a lottery ticket and watching a sporting event [40]. Examples of single sentence practice and conversational script practice from the AphasiaScripts® software are shown in Fig. 4.

### Blinding

In order to achieve blinding, a speech-language pathologist who is not directly associated with protocol administration and blind to the group assignment will administer and score all the assessments. Participants will also be blind to the timing of tDCS. In sham tDCS, the direct current is applied for 30 s and then the tDCS is switched off. The 30 s of stimulation does not induce any neural or behavioral changes, but participants feel the sensations associated with tDCS. In the online tDCS arm, sham tDCS will be applied at the beginning or end of the session. There is also a sham tDCS arm in which participants will not receive tDCS; they will receive only 40 min of SLT.

### Data management and confidentiality

The data collected from the project will not be part of the subject’s clinical files. We will de-identify personal information and use codes instead. The computer files will be encrypted and password protected while data files, disks, and reports will be physically secured in a locked area with only authorized personnel having access to them. A master sheet with subject’s name and contact information will have to be maintained separately to enable initial review of their clinical information and contact throughout the study. De-identified data may be stored indefinitely. Records with identifiable data will be destroyed at the end of the study. Only authorized personnel listed on this IRB will have access to the data.

### Study endpoints

#### Primary outcomes

A simulated conversation (i.e., conversation with a speech-language pathologist) of the trained script will be recorded three times at baseline, immediately post-treatment, and at the 4- week and 8-week follow-up visits. The primary outcome measure is change in accuracy and rate of production on the trained script during a simulated conversation from pre- to immediately post-treatment.

#### Secondary outcomes

Secondary outcomes will be change from baseline to 4- and 8-week follow-up visits on the trained conversational script. We will also measure performance on an untrained script which will be matched for length and complexity using the Flesch-Kincaid readability index [40]. The untrained script will be used to assess generalization from pre- to post-treatment. Secondary outcomes will also include the change in WAB-R AQ from baseline to post-treatment.

### Statistical analysis

The primary outcome will be the change in performance (accuracy) from baseline to post-treatment on the trained script during a simulated conversation. Secondary outcomes will be (1) change in performance from baseline to 4 and 8 weeks post-treatment on the trained script during a simulated conversation, to assess maintenance, and (2) change in performance from baseline to post-treatment and baseline to 4 and 8 weeks post-treatment on the untrained script to assess generalization. Descriptive statistics (mean, SD, and 95% confidence intervals) will be used to summarize the data by groups. In addition, these outcomes will be analyzed using a two-sample *t*-test comparing changes from baseline to post-treatment between two treatment arms. The selected treatment arm will be compared to the sham tDCS arm. Paired *t*-tests will be used to determine whether changes in outcome persist at 4 and 8 weeks post-treatment persist (i.e., are they equal to 0). A more comprehensive analysis will be done using a mixed effects model which is robust to missing data to analyze changes over time.

### Safety

The most often reported effects of tDCS are tingling and itching sensations under the electrodes, headaches, and tiredness [43]. These tDCS-induced sensations of tingling and itching can be associated with salinity of the solution used for electrode sponges [43]. Our electrode sponges will be soaked in a solution with appropriate NaCI solution concentration of 15 and 140mM [44]. We will inject saline into the electrode sponges periodically to make sure they are not dry. This will ensure that tDCS-induced discomforts due to dry electrode sponges are minimized [45]. In addition, self-reported tDCS-induced sensations will be obtained using aphasia-friendly questionnaires. The same questionnaires will be administered at the follow-up sessions. If discomfort is reported, we will assess whether the discomfort is common to tDCS (e.g., itching and tingling) and then make sure if the appropriate amount of saline is applied to the electrodes. We will also make a determination of the severity of tDCS-induced discomfort when it is brought to our attention. If we determine that these unanticipated effects are not tolerable for the participant, we will terminate the experiment.

## Discussion

This protocol describes a randomized clinical trial to investigate the timing of tDCS in combination with SLT for individuals with aphasia. This study is motivated by evidence for tDCS as a potential adjuvant to SLT [15, 16, 18]. Existing studies of tDCS in combination with SLT apply tDCS concurrently with SLT. It is presumed that performance improvements following online vs. offline tDCS are driven by different processes [26–29]. The objective of the current study is to investigate the role of timing parameters of tDCS relative to SLT. To our knowledge, our study is the first to evaluate the timing of tDCS relative to SLT for aphasia treatment.

Identifying the optimum timing to combine tDCS with SLT can guide treatment approaches to facilitate better outcomes for people with aphasia. Furthermore, the protocol described here uses fMRI-guided neuronavigation to identify the optimal site to apply tDCS electrodes. Locating the stimulation site in this way allows the specific lesion characteristics of each participant to be taken into account. A potential contribution of this approach is that it may help address issues of variability in other studies, where lesion site is variable but placement of tDCS electrodes is the same across participants [6].

The SLT used in this study uses a simulated real-world conversation. As such, the study may extend knowledge of tDCS in aphasia therapy beyond naming or word-level practice into the area of connected discourse. Lastly, the effect sizes derived from this study related to the particular treatment arm may guide other tDCS trials that investigate the impact of tDCS on functional communication in individuals with aphasia.

## Dissemination

Results from the study will be published in peer-reviewed journals and academic conferences.

## Trial status

The protocol version is 10.0 (2021). Recruitment began in 2018 and is expected to be completed by 2023.

## Data Availability

Data is available upon request at the end of the trial from the corresponding author.

## Abbreviations

PWA: Persons with aphasia
tDCS: Transcranial direct current stimulation
SLT: Speech-language therapy
WAB-R-AQ: Western Aphasia Battery-Revised-Aphasia Quotient
AG: Angular gyrus
pSTG: Left posterior superior temporal gyrus
IFG: Inferior frontal gyrus
LTP: Long-term potentiation
BCM: Bienenstock-Cooper-Munro
